# Seasonal variation in the detection rate and all-cause in-hospital mortality of AKI in China: A nationwide cohort study

**DOI:** 10.3389/fpubh.2022.947185

**Published:** 2022-10-03

**Authors:** Jiaqi Li, Qingqing Zhou, Daoning Zhang, Jinwei Wang, Li Yang

**Affiliations:** ^1^Renal Division, Department of Medicine, Peking University First Hospital, Beijing, China; ^2^Institute of Nephrology, Peking University, Beijing, China; ^3^Research Units of Diagnosis and Treatment of Immune-Mediated Kidney Diseases, Chinese Academy of Medical Sciences, Beijing, China

**Keywords:** acute kidney injury (AKI), seasonality, detection rate, in-hospital mortality, epidemiology

## Abstract

**Background:**

Acute kidney injury (AKI) is a severe clinical syndrome that places a massive burden on medical systems worldwide, yet the seasonality of AKI remains unexplored in China. The aim of this study was to describe the seasonal variation in the detection rate and all-cause in-hospital mortality of AKI in China based on a nationwide cohort study.

**Methods:**

This was a retrospective cohort recruiting a national sample of 7,291 adult patients treated in hospitals in 22 provinces of mainland China during January or July 2013. AKI was defined according to the 2012 Kidney Disease Improving Global Outcomes AKI creatinine criteria or expanded criteria of increase or decrease in serum creatinine level of 50% during the hospital stay. The seasonal group was determined according to the corresponding admission date for each patient. The detection rate of AKI refers to the ratio of identified AKI cases to the total number of adult admissions from the same regional or seasonal group.

**Results:**

Both the detection rate (2.31 vs. 2.08%, *p* = 0.001) and in-hospital mortality rate (13.3 vs. 10.7%, *p* = 0.001) of AKI were higher in winter than in summer. The patients with AKI detected in winter had higher proportions of prehistory diseases, cardiac or vascular kidney injury factors, and severe comorbidities than those in summer (all *p* < 0.05). In the multivariable analysis, winter was an independent risk factor for in-hospital mortality of patients with AKI [odds ratio (OR) = 1.22, 95% confidence interval (CI), 1.03–1.44, *p* = 0.02] after adjusting for demographic factors, medical history, comorbidity, and climatic confounders. Higher ambient temperature (OR = 0.91, 95% CI, 0.86–0.97, *p* = 0.002, per 10°C increase), higher relative humidity level (OR = 1.14, 95% CI, 1.04–1.25, *p* = 0.005, per 10% increase), and living in temperate continental region (OR = 2.18, 95% CI, 1.63–2.91, *p* < 0.001) were each independently associated with in-hospital mortality.

**Conclusion:**

The detection rate and all-cause in-hospital mortality of AKI showed a winter predominance in patients with AKI in China. Winter appeared to be an independent risk factor for all-cause in-hospital mortality in patients with AKI. Environmental factors, including lower ambient temperature, higher relative humidity level, and living in temperate continental climatic regions, were each independently associated with increased risks of in-hospital mortality in patients with AKI.

## Introduction

Acute kidney injury (AKI) is a clinical syndrome characterized by a sudden deterioration of kidney function (1). It is a severe but common complication in hospitalized patients. Approximately 10–15% of all hospitalized patients (2) and over 50% of patients in the intensive care unit (ICU) suffer from AKI (3). The high morbidity and mortality of AKI cause a huge burden on the medical system worldwide (4, 5). In response to the global target of “0 by 25” launched by the International Society of Nephrology (ISN), Chinese experts conducted a national survey on the epidemiology of AKI in China and reported several challenges in the recognition, diagnosis, and management of AKI nationwide (6).

Understanding the seasonal pattern of AKI would contribute to the improvement of clinical management and prevention care. Previous studies have demonstrated a remarkable increase in hospitalization and mortality in winter related to cardiovascular, respiratory, and infectious diseases (7–11). Increased sympathetic activity and elevated blood pressure were proven to be a result of cold temperature (12). In summer, higher levels of serum creatine and lower levels of eGFR were observed (13). Despite the remarkable trend, only a few studies have focused on the seasonal pattern of AKI (14). Experts from Wales, Japan, and Italy illustrated that the occurrence of AKI was highest in winter among all seasons and that the disease showed higher severity and worse clinical prognosis in winter (15–17). Although underlying diseases with winter predominance could precipitate AKI and result in worse renal function recovery (14), environmental factors, including ambient temperature and relative humidity level, were also found to correlate with an increased risk of AKI independently (17, 18).

At present, the seasonal pattern of AKI in China remains unexplored. As climate patterns differ across different parts of the world, it is difficult to synthesize the seasonality of AKI. Therefore, we explored the seasonal variation in the detection rate and outcome of AKI among hospitalized patients using a large database of patients with AKI from 22 provinces in mainland China. We also examined the association of environmental factors, including average temperature, relative humidity level, and climate type, with all-cause in-hospital mortality in patients with AKI.

## Methods

### Study design and population

**Figure 1:**
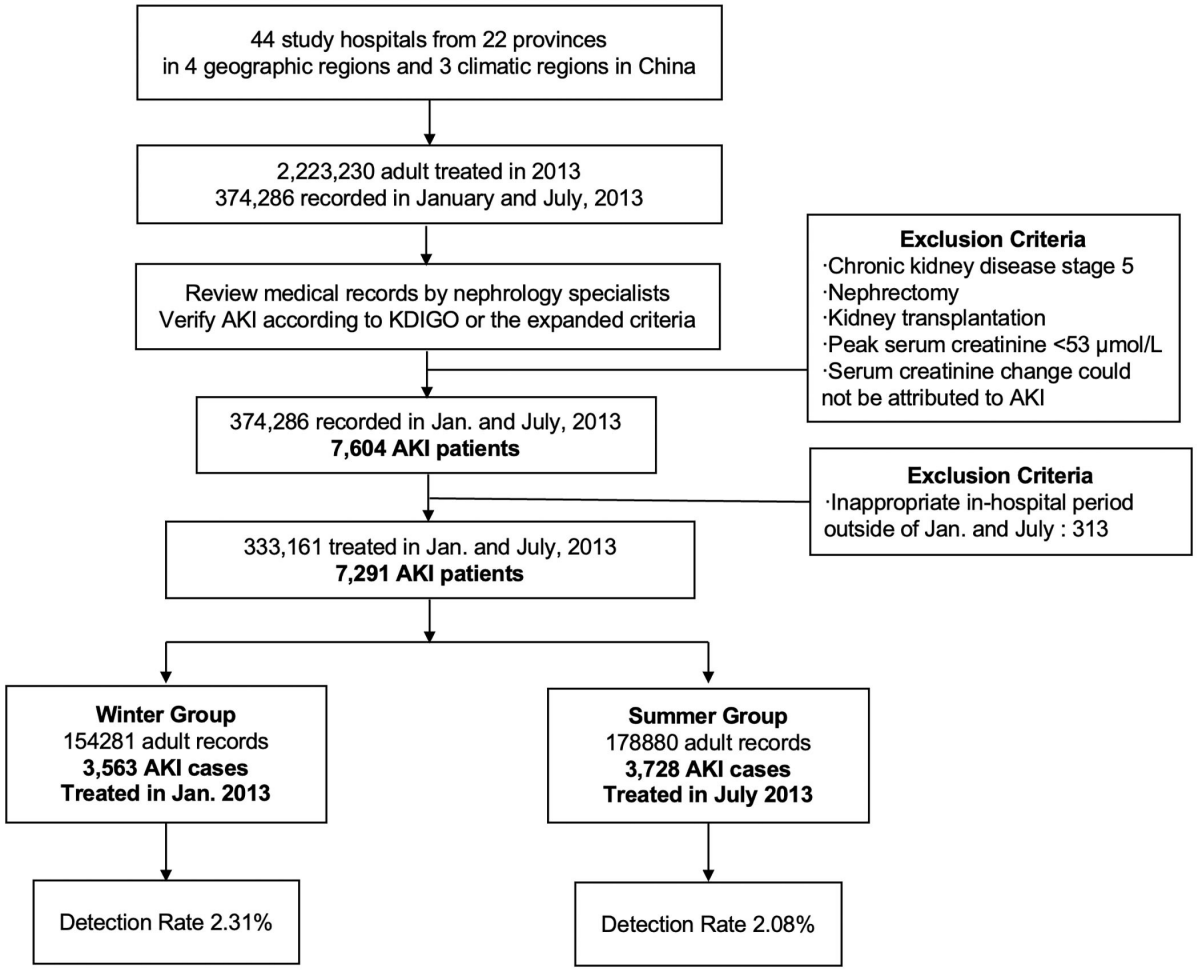
The flowchart of study participants.

The study was approved by the Ethics Committee of Peking University First Hospital (2014) (6). The survey protocol had previously been described in detail (6). A study design flowchart is presented in Figure 1. Altogether, a total of 2,223,230 hospitalized adult patients from 22 academic hospitals and 22 local hospitals in 22 provinces of mainland China in 2013 were screened. Considering the workload of reviewing medical records, 374,286 adult patients recorded in January and July 2013 were selected to represent winter and summer, respectively. Nephrology specialists screened each record to verify the diagnosis of AKI. The identification criteria of AKI included (1) the 2012 Kidney Disease: Improving Global Outcomes definition of AKI (KDIGO criteria): an increase in serum creatine (Scr) level by 0.3 mg/dl within 48 h or by 50% within 7 days (not including urine output criteria); and (2) expanded criteria for patients with no repeated serum creatinine assay within 7 days or with recovering AKI: an increase or decrease in Scr level of 50% during the hospital stay (6, 19). Patients meeting the exclusion criteria (a. chronic kidney disease stage 5; b. nephrectomy or kidney transplantation; c. peak Scr < 53 μmol/L; and d. Scr change could not be attributed to AKI) were removed from the study.

In total, 7,604 patients with AKI from 374,286 adult patients recorded in January and July of 2013 were identified. We further retrieved the medical records to check the admission date and hospitalization period for each patient. Most of the patients recorded in January or July of 2013 were admitted in the same month, with a small proportion admitted earlier in December 2012 (7.8%) or June 2013 (13.5%), respectively. Other patients hospitalized outside the appropriate period were removed from the study population (*n* = 313), leaving 7,291 patients with AKI (3,563 in winter and 3,728 in summer).

### Data collection

In the survey, demographic characteristics, the season of admission to hospitalization, hospital type, medical history, cause of AKI, injury factors of AKI, comorbid conditions, peak AKI stage (1–3 according to KDIGO criteria), length of hospital stay, all-cause in-hospital mortality, renal replacement therapy (RRT) indication, and renal recovery at discharge for each patient were collected. Injury factors were recorded when patients had the onset of related kidney problems. Hypovolemia included the onset of dehydration caused by vomiting, diarrhea, heatstroke, gastrointestinal hemorrhage, severe acute pancreatitis, and rhabdomyolysis. Renal vascular constriction was recorded when patients had renal toxicity injuries or used vasoconstriction medications. Mechanic obstruction was recorded when patients had severe postrenal AKI caused by urinary lithiasis or compression of hematoma.

We further classified the patients into two different types of AKI [hospital-acquired (HA) or community-acquired (CA)]. CA-AKI was identified according to the following criteria (19): (1) the level of Scr increased at admission and decreased during hospitalization; (2) the level of Scr increased at admission and continued to increase or remained at a high level during hospitalization; or (3) renal function was normal at admission, but the Scr level rose to the level that could be defined as AKI within 2 days after hospitalization. The rest of the patients were classified as HA-AKI.

### Environmental and social variables

The data on average temperature (°C) in January 2013 and July 2013 were obtained from a database named “Monthly Surface Temperature in China 0.5° × 0.5° Grid Dataset (V2.0)”, which was established by the National Meteorological Information Center (NMIC; ID: 1.2.156.416.CMA.D3-S.202101.QAB1M). The monthly average temperature was matched with the exact latitude and longitude location where patients with AKI were hospitalized, which was accurate to 0.5°. The data of monthly average relative humidity levels at the city level were obtained from an NMIC database named the “Monthly Climatological Dataset of China Ground International Exchange Station” (ID: 1.2.156.416.CMA.D3.A002.001.OB.WB.CHN.MUL.MON.STA.1).

As China is a country with a vast territory, we stratified mainland China into four geographical regions based on latitude and longitude (north, northwest, southeast, and southwest), as well as three climatic regions (temperate monsoon, temperate continental, and subtropical monsoon). In terms of the economic development level, the provinces involved were divided into three groups (high, middle, and low) based on their ranking of yearly per capita GDP, according to the data from the 2013 report of the National Health and Family Planning Commission of the People's Republic of China and the National Bureau of Statistics of the People's Republic of China (22).

### Study outcomes

The detection rate of AKI refers to the ratio of identified AKI cases to the total number of adult admissions from the same regional or seasonal group. The follow-up period started on the admission date for the patients hospitalized just during January or July but on 1 January 2013 or 1 July 2013 for the patients admitted preceding the index months, while all patients ended follow-up on the date of either death or discharge from hospital. The all-cause in-hospital mortality rate of AKI was defined as the ratio of identified deaths during hospitalization to the total number of hospitalized patients with AKI. The primary exposures were environmental factors, including season (winter or summer), ambient temperature, relative humidity level, and climatic regions.

### Statistical analysis

Categorical variables were described as frequencies (percentages). Continuous variables were described as the mean (standard deviation, SD) or median (interquartile range, IQR), as appropriate. Comparisons between groups were made by using Fisher's exact test for categorical variables and the Kruskal–Wallis test for continuous variables. The detection rate was examined using the chi-squared test. A multivariable logistic regression model was used to analyze the relationship between the exposure variables [climate type, monthly average environmental temperature (per 10°C), and relative humidity level (per 10%)] and in-hospital mortality. Covariates were selected by backward stepwise selection using the Akaike information criterion (AIC), including age, sex, history of chronic diseases (hypertension, cardiac dysfunction, diabetes mellitus, and tumor), injury factors (prerenal and postrenal factors), comorbidities (ARDS, sepsis, and MODS), AKI stage at peak (Stages 1–3), AKI types (HA- or CA-AKI), climatic regions, and GDP levels, except when climatic regions themselves were treated as the primary exposure. Two multivariable analysis models were established. In Model 1, environmental exposures were added one at a time with the aforementioned covariates adjusted. In Model 2, a fully adjusted model was established and thus treated as the main analysis, with ambient temperature and relative humidity additionally added as covariates, except when the factors themselves were treated as the main exposure. Odds ratios (ORs) with 95% confidence intervals (CIs) and *p-*values of the Wald χ^2^ test were reported. The data were analyzed and managed with R (version 3.6.1) within the R studio platform (version 1.3.1093). All *p*-values were two-sided, and *p* < 0.05 was considered statistically significant.

## Results

### Seasonal variation in the detection rate of AKI

**Figure 2:**
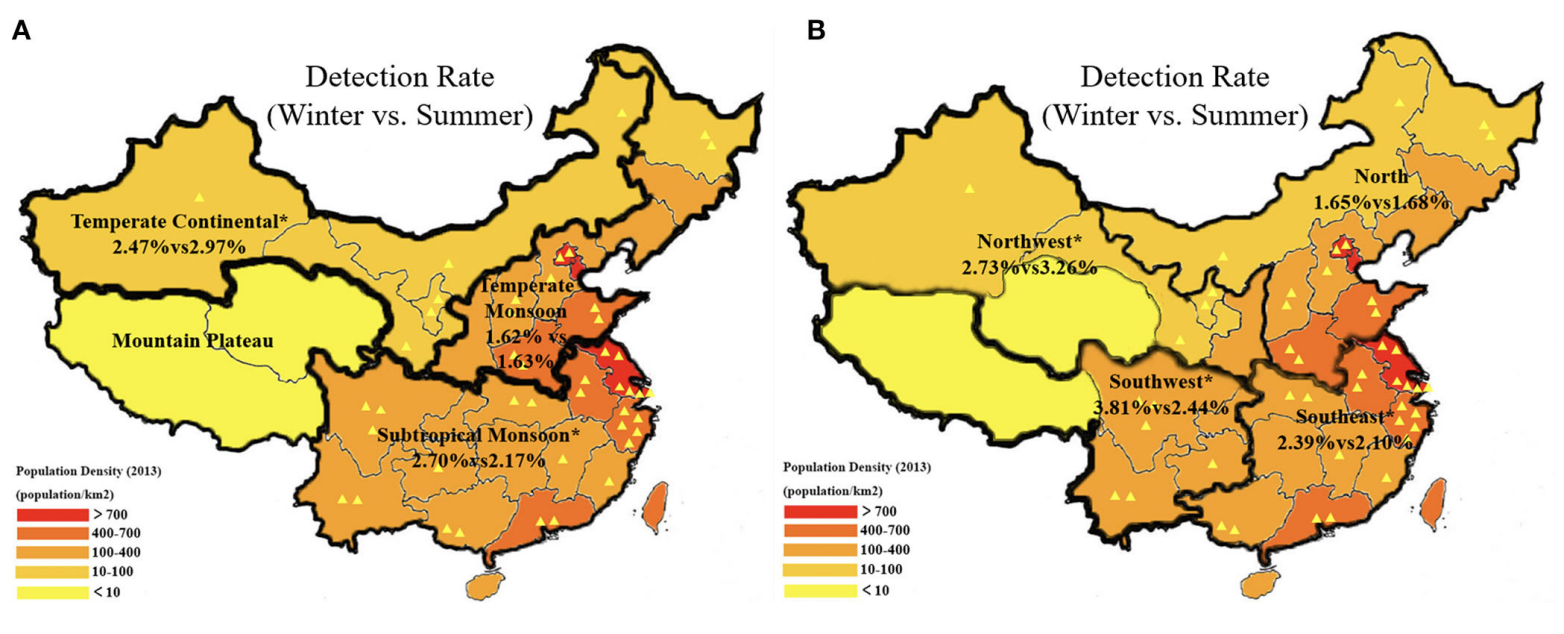
The detection rates of AKI during winter and summer in different geographical and climatic regions. **(A)** The detection rate (winter vs. summer) of all-type AKI in 3 climatic regions, namely, temperate continental, temperate monsoon, and subtropical monsoon; **(B)** The detection rate (winter vs. summer) of all-type AKI in 4 geographical regions, namely, North, Northwest, Southeast, and Southwest. The population density is marked with different colors. Each yellow triangle represents a study hospital. *p < 0.05.

Among the 333,161 patients recorded in the hospital during either of the 2 seasons studied (winter or summer), 7,291 cases were identified as having AKI. Among them, 3,563 patients were treated in January 2013, and the other 3,728 patients were treated in July 2013. A total of 3,928 patients were identified as having CA-AKI, and the other 3,309 patients were classified as having HA-AKI. The detection rate of all-type AKI in winter hospitalization was 2.31% (3,563 of 154,281; Table 1), which was significantly higher than that of 2.08% (3,728 of 178,880) in summer (*p* = 0.001). The detection rates of both CA-AKI and HA-AKI were also higher in winter than in summer (1.24 vs. 1.16%, *p* = 0.03; 1.07 vs. 0.93%, *p* = 0.001). The winter predominance of the detection rate of all-type AKI can be observed across climatic regions, hospital types, geographical regions, and economic development levels, except for northwest and temperate continental regions, where the detection rate was higher in summer (2.73 vs. 3.26% *p* = 0.01; 2.47 vs. 2.97% *p* = 0.01; Figure 2).

**Table 1:** Comparison of detection rates in patients with different seasons.

	**Winter**			**Summer**			***p*-value**
	**Total**	**No. of cases**	**Rates**	**Total**	**No. of cases**	**Rates**	
All-type AKI	154281	3563	2.31%	178880	3728	2.08%	0.001
CA-AKI	154281	1913	1.24%	178880	2073	1.16%	0.03
HA-AKI	154281	1650	1.07%	178880	1655	0.93%	0.001
**Hospital type**
Academic	100309	2587	2.58%	119397	2832	2.37%	0.02
Local	53972	976	1.81%	59483	896	1.51%	< 0.001
**Region**
North	57516	947	1.65%	62300	1044	1.68%	0.71
Northwest	13424	366	2.73%	13948	455	3.26%	0.01
Southeast	65271	1562	2.39%	80664	1693	2.10%	< 0.001
Southwest	18070	688	3.81%	21969	536	2.44%	< 0.001
**Climate**
Temperate monsoon	51481	832	1.62%	57150	931	1.63%	0.88
Temperate continental	19459	481	2.47%	19097	568	2.97%	0.003
Subtropical monsoon	83341	2250	2.70%	102633	2229	2.17%	< 0.001
**Per capita GDP levels**
Low	42473	1155	2.72%	49522	1045	2.11%	< 0.001
Medium	60204	1349	2.24%	66184	1598	2.41%	0.04
High	51604	998	1.93%	63175	1085	1.72%	0.01

HA-AKI, hospital-acquired acute kidney injury; CA-AKI, community-acquired acute kidney injury.

### Characteristics of patients with AKI in different seasons

The mean age of all-type patients with AKI was 61.5 ± 18.9 years of age, and 65% of the patients were men (Table 2). Patients hospitalized in winter were older (62.5 ± 18.1 vs. 60.0 ± 17.0, *p* < 0.001) and had higher proportions of prehistory diseases than those in summer, including cardiovascular diseases, hypertension, and CKD history (all *p* < 0.05). Prerenal factors were the major cause of AKI in both seasons, with a slightly higher proportion in winter (72.6 vs. 70.2%, *p* = 0.02). By comparison, the proportion of postrenal AKI was lower in winter than in summer (7.5 vs. 10.2%, *p* < 0.001). Injury factors of the kidney, including cardiac dysfunction, vascular dilation, renal vascular constriction, and sepsis, were much more common in winter than in summer (all *p* < 0.05). Severe comorbidities, including sepsis, ARDS, and shock, also occurred more frequently in winter (all *p* < 0.05). The all-cause in-hospital mortality rate among all AKI cases was 11.8% (860 of 7,291), and a higher mortality rate was discovered in winter than in summer (13.3 vs. 10.7%, *p* = 0.001). A significantly lower rate of recovery at discharge (62.5 vs. 69.5%, *p* < 0.001) was also observed in winter.

**Table 2:** Comparison of demographic and clinical characteristics of patients with AKI in different seasons.

**Number of patients**	**Total**	**Winter**	**Summer**	***p*-value**
	***N* = 7291**	***N* = 3563**	***N* = 3728**	
**Demographics^1^**
Age, years	61.5 ± 18.9	62.5 ± 18.1	60.0 ± 17.0	< 0.001
Male	4742 (65.0%)	2277 (63.9)	2465 (66.1)	0.04
**General type of AKI**				0.12
Hospital-acquired AKI	3309 (45.4)	1650 (46.3)	1659 (44.5)	
Community-acquired AKI	3982 (54.6)	1913 (53.7)	2069 (55.5)	
**Medical history^3^**
Previous CVD	2006 (27.5)	1052 (29.5)	954 (25.6)	< 0.001
Previous HT	3046 (41.8)	1544 (43.3)	1502 (40.3)	0.008
Previous DM	1341 (18.3)	672 (18.9)	669 (17.9)	0.31
Preexisting CKD	1768 (24.3)	944 (26.5)	824 (22.1)	< 0.001
Malignancy	1331 (18.3)	624 (17.5)	707 (19)	0.11
**Cause of AKI**
Prerenal	5205 (71.4)	2588 (72.6)	2617 (70.2)	0.02
Intrinsic renal	2089 (28.7)	1045 (29.3)	1044 (28)	0.21
Postrenal	647 (8.9)	266 (7.5)	381 (10.2)	< 0.001
**Injury factors**
Hypovolemia	3780 (51.8)	1859 (52.2)	1921 (51.5)	0.58
Cardiac dysfunction	2054 (28.2)	1061 (29.8)	993 (26.6)	0.003
Vascular dilation	2380 (32.6)	1209 (33.9)	1171 (31.4)	0.02
Renal vascular dysfunction	888 (12.2)	479 (13.4)	409 (11)	< 0.001
Mechanic obstruction	118 (1.6)	60 (1.7)	58 (1.6)	0.67
Nephrotoxicity	5236 (71.8)	2562 (71.9)	2674 (71.7)	0.87
Sepsis	453 (6.2)	246 (6.9)	207 (5.6)	0.02
**AKI stage at peak**				0.21
1	3381 (46.4)	1660 (46.6)	1721 (46.2)	
2	1872 (25.7)	938 (26.3)	934 (25.1)	
3	2038 (28)	965 (27.1)	1073 (28.8)	
**Comorbidities**
MODS	888 (12.2)	455 (12.8)	433 (11.6)	0.13
ARDS	419 (5.7)	228 (6.4)	191 (5.1)	0.02
Sepsis	453 (6.2)	246 (6.9)	207 (5.6)	0.02
Shock	860 (11.8)	451 (12.7)	409 (11)	0.03
DIC	75 (1)	30 (0.8)	45 (1.2)	0.12
**Treatment of AKI**			
Specialist consultant	1527 (21)	723 (20.3)	804 (21.6)	0.18
RRT	512 (7)	261 (7.3)	251 (6.7)	0.32
RRT indication	829 (11.4)	417 (11.7)	412 (11.1)	0.38
**Length of follow-up**	19.5 ± 18.9	20.3 ± 21.1	18.7 ± 16.4	0.08
**In-hospital mortality^2^**	860 (11.8)	468 (13.3)	392 (10.7)	0.001
**Renal prognosis**
Recovery	4737 (65)	2203 (62.5)	2534 (69.5)	< 0.001
Recurred	344 (4.7)	163 (4.7)	181 (5.1)	0.37
Maintenance RRT	185 (2.5)	110 (3.1)	75 (2.0)	0.01

1 The data are presented either as the mean ± SD, median (IQR), or n (%).

2 A total of 120 patients (40 in the winter group and 80 in the summer group) missed data on in-hospital morality.

3 AKI, acute kidney injury; CVD, cardiac vascular disease; HT, hypertension; DM, diabetes mellitus; CKD, chronic kidney disease; RRT, renal replacement treatment; Scr, serum creatinine; MODS, multi-organ damage syndrome; ARDS, acute respiratory distress syndrome; DIC, disseminated intravascular coagulation.

### Association of seasonal and environmental factors with all-cause in-hospital mortality

**Figure 3:**
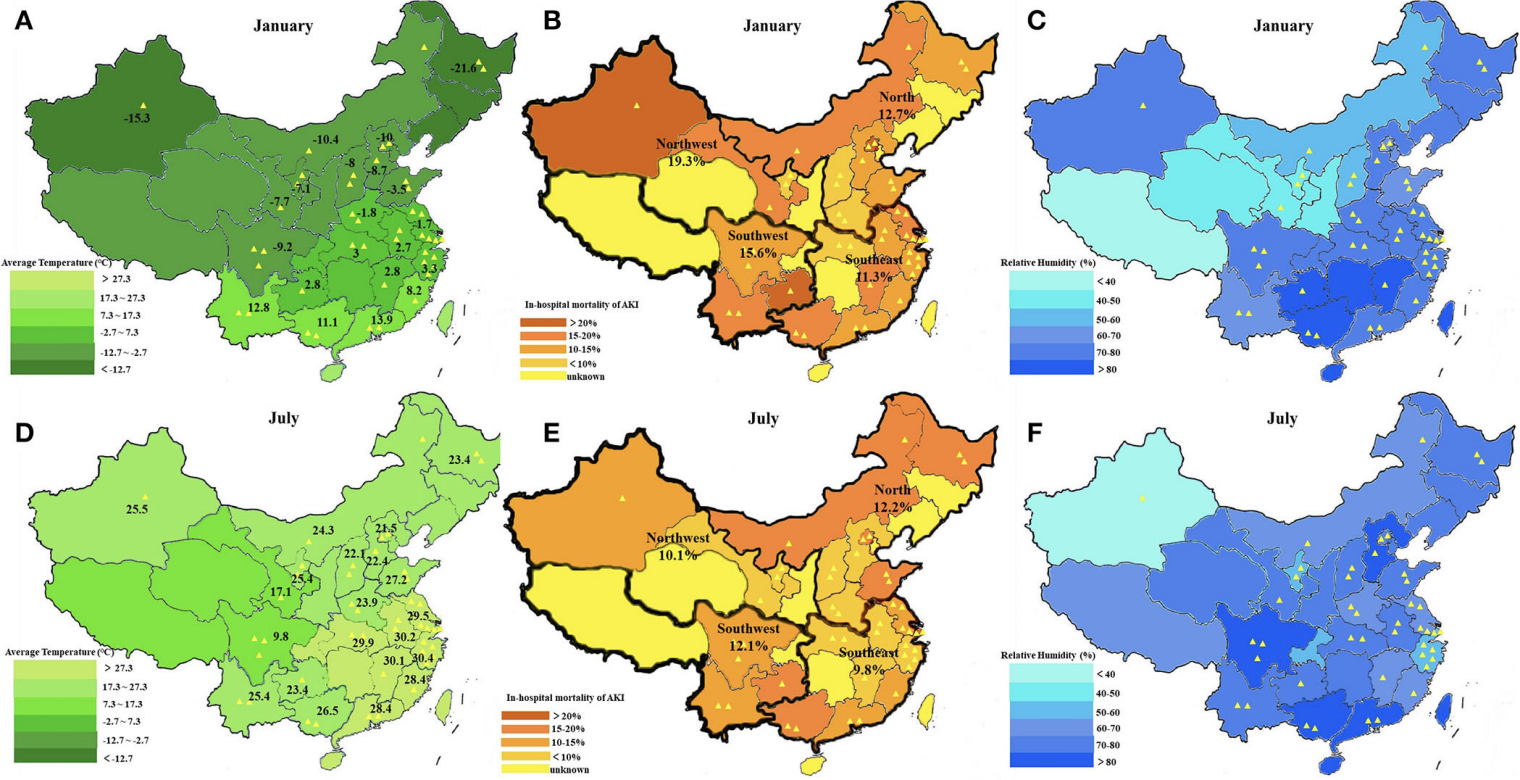
Map of monthly average temperature and relative humidity level in January and July 2013 across different cities in China. **(A)** The monthly average temperature in January; **(B)** In-hospital mortality of patients with AKI in January; **(C)** The relative humidity level in January; **(D)** The monthly average temperature in July; **(E)** In-hospital mortality of patients with AKI in July; and **(F)** The relative humidity level in July. Each yellow triangle represents a study hospital.

We then examined the impact of seasonal and environmental factors on all-cause in-hospital mortality in all-type patients with AKI. A total of 7,171 patients with complete records of in-hospital mortality information were used in the analysis. Those excluded because of missing information on outcomes involved more men and more patients hospitalized in summer, had lower proportions of preexisting CKD, intrinsic-renal cause of AKI, and nephrotoxicity factor, but had higher proportions of AKI stage 3, MODS, and RRT, compared with those included in the analysis, thus potentially representing a higher risk profile (Supplementary Table S1). The overall distribution of the in-hospital mortality rate, levels of average temperature, and relative humidity across different regions and provinces in the country are shown in Figure 3. The multivariable logistic regression model adjusted for age, sex, medical history of chronic diseases, injury factors, comorbidities, AKI stage at peak, types of AKI (HA- or CA-AKI), climatic regions, and GDP levels (Table 3) revealed that winter (vs. summer) was an independent risk factor for all-cause in-hospital mortality of AKI (OR = 1.22, 95% CI: 1.03–1.44, *p* = 0.02). The risk of in-hospital mortality decreased by 11% per 10°C increase in the average monthly ambient temperature (OR = 0.89, 95% CI: 0.84–0.94, *p* = 0.001) and increased by 19% per 10% increase in the local relative humidity level (OR = 1.19, 95% CI: 1.09–1.29, *p* = 0.001). Patients living in temperate continental regions had a higher risk of in-hospital mortality than those in subtropical monsoon areas (OR = 1.89, 95% CI: 1.50–2.38, *p* < 0.001). In the multivariable logistic regression model fully adjusted for environmental factors, with ambient temperature and relative humidity level serving as an adjusted covariate for each other, respectively, both average temperature per 10°C increase (OR = 0.91, 95% CI: 0.86–0.97, *p* = 0.002) and relative humidity level per 10% increase (OR = 1.14, 95% CI: 1.04–1.25, *p* = 0.005) were independently associated with the all-cause in-hospital mortality of AKI.

**Table 3:** Multivariable logistic regression model analysis of environmental factors associated with in-hospital mortality of patients with AKI.

**Variables**	**Crude OR (95%CI)**	**Model 1^1^^,^ ^3^** **adjusted OR (95% CI)**	***p*-value**	**Model 2^2^** **adjusted OR (95% CI)**	***p*-value**
**Season**
Summer	Reference	Reference		Reference	
Winter	1.27 (1.10, 1.46)	1.22 (1.03, 1.44)	0.02	NA	NA
Average temperature (per 10°C increase)	0.89 (0.85, 0.93)	0.89 (0.84, 0.94)	0.001	0.91 (0.86, 0.97)	0.002
Relative humidity (per 10% increase)	1.05 (0.99, 1.13)	1.19 (1.09, 1.29)	0.001	1.14 (1.04, 1.25)	0.005
**Climate**
Subtropical monsoon	Reference	Reference		Reference	
Temperate monsoon	1.03 (0.86, 1.22)	0.94 (0.76, 1.15)	0.53	0.96 (0.78, 1.19)	0.70
Temperate continental	1.31 (1.08, 1.59)	1.89 (1.50, 2.38)	< 0.001	2.18 (1.63, 2.91)	< 0.001

1 **Model 1** adjusted for age, sex, prehistory diseases (hypertension, cardiac dysfunction, diabetes mellitus, and tumor), injury factors (prerenal and postrenal factors), comorbidities (ARDS, sepsis, and MODS), AKI stage at peak (Stages 1–3), AKI types (HA-or CA-AKI), climatic regions, and GDP levels, except when climatic regions themselves were treated as the main exposure.

2 **Model 2** included all factors in Model 1 plus both average ambient temperature and relative humidity level for the analysis of climatic regions, average temperature for relative humidity, and relative humidity for average temperature.

3 A total of 120 patients (40 in the winter group and 80 in the summer group) missed the relevant data on in-hospital morality, leaving 7,171 patients included in the analysis.

## Discussion

Our study describes the seasonal variation in the detection rate and in-hospital mortality of AKI between winter and summer in China and analyzes the association of seasonal and environmental factors with the all-cause in-hospital mortality of patients with AKI.

The detection rate and all-cause in-hospital mortality of AKI showed a winter predominance in China. Prerenal AKI was more prevalent in winter, while the proportion of postrenal AKI was higher in summer. Higher proportions of patients had prehistory diseases, cardiac and vascular kidney injury factors, and severe comorbidities in winter, which partially explained the “winter peak”. Winter was an independent risk factor for in-hospital mortality in patients with AKI. Lower ambient temperature and higher humidity levels increased the risk of in-hospital mortality in patients with AKI.

Seasonal changes in AKI have been studied across different regions. The first study from Wales reported that the detected number of patients with AKI peaked in the first 3 months of a year and fell during each quarter of the year, which was likely attributed to the seasonal pattern of primary illnesses related to kidney injury (16). A later study from Japan reported an increase in local AKI incidence with a higher mortality rate during winter after adjusting for demographic factors, comorbidities, and medications, which indicated that season was independently associated with the incidence and outcome of AKI (15). The latest study from Italy also reported a “winter peak” of AKI in an Italian hospitalized cohort, and they further revealed that environmental factors, including average ambient temperature and relative humidity level, were also independently associated with the incidence of AKI (17). Consistent with previous investigations, our study also reported a winter predominance in the detection rate and in-hospital mortality of AKI, and to the best of our knowledge, we might be among the first to reveal that seasonal and environmental factors were independently associated with the in-hospital mortality of patients with AKI. These findings support the hypothesis that seasonal variation may exist as far as the occurrence and outcome of patients with AKI are concerned.

As China is a country with a vast territory covering several different climate patterns, we analyzed the detection rate of AKI stratified by geographical and climatic regions. A winter predominance in the detection rate of AKI was shown in southeastern and southwestern China, but an opposite trend was found in northwestern China. Subtropical and temperate monsoon climatic regions showed consistent winter predominance; however, a summer predominance was manifested in temperate continental regions. This exception was closely related to the high incidence of postrenal AKI, as the hot weather in the northwest during summer (21) increased the incidence of dehydration and lithogenesis (18, 23–27). Studies also reported that the prevalence of urinary stone disease in several provinces in southern China (10–12%) was higher than average in summer (19, 21). However, the detection rate of AKI in southern and subtropical areas in summer was still inferior to that in winter.

The prognosis of patients with AKI in winter was much worse than in summer, with a lower recovery rate, higher recurrence and RRT maintenance rate, and higher mortality rate, which was in agreement with previous studies (15, 16). The deterioration of underlying diseases precipitating AKI in winter (cardiovascular and respiratory diseases) and the higher occurrence of related comorbidities (sepsis) could partly explain the poor prognosis in winter (28). However, after adjusting for potential confounders (demographic factors and comorbidities), we revealed that in winter, environmental factors, including ambient temperature, relative humidity, and climatic zone, were each independently associated with in-hospital mortality.

The discovery of higher temperatures resulting in a lower risk of mortality indicated that a warm ambient environment could be more beneficial for renal function recovery in patients with AKI during their hospital stay. One possible explanation could be that the prevalence of postrenal AKI in summer improved the overall prognosis of hospitalization after treatment with fluid therapy and lithotripsy (20). In addition, changes in vascular function and activity of the sympathetic nervous system in cold winter might influence the recovery process and lead to adverse outcomes (12). More importantly, we discovered that the ambient temperature remained an independent factor after adjusting for humidity level, which was inconsistent with the previously mentioned Italian study (17). Based on several findings of ambient temperature changing the physiologic and pathologic progress of AKI (24, 29), we prefer to stress the importance of temperature on the clinical outcome of patients with AKI.

Regarding humidity, the aforementioned Italian study demonstrated that a high humidity level was an independent predictor for AKI incidence after adjusting for temperature factors (17), yet they did not explore the association with the outcome of patients with AKI. We discovered that a high humidity level was an independent risk factor for in-hospital mortality. Although the mechanism of how humidity affects health and diseases remains elusive, previous studies proved that anomalous mortality increased in humid areas because humidity had an impact on cold or heat stress and the hydration state of the human body (30). Loss of salt and water in high humidity conditions resulted in hemoconcentration and increased blood viscosity, placing strain on various systems and leading to a higher mortality rate (31), which could explain the poor outcome of patients with AKI in regions with higher humidity. In addition, previous studies reported that a combined effect of high humidity and low temperature exacerbated preexisting cardiac health problems and led to the highest mortality of cardiovascular diseases. In contrast, the combination of low humidity and the high temperature had the lowest mortality of cardiovascular diseases (32–34). As both higher ambient temperature and lower relative humidity level were identified in our study to be independently associated with lower in-hospital mortality, we would suppose the same coeffect of the environmental factors works on the recovery of renal function, which should be given greater attention in the clinical management and prevention of AKI.

The advantage of our study lies in its nationwide multicenter sample of patients with AKI. The limitations of the study lie in its retrospective design, lack of an all-population database, unevenness in the number of patients in different regions, and lack of spring and autumn in the description of seasonal patterns, which warrant further investigation. Nevertheless, we assume that the influence of environmental factors could be clearer when comparing seasons with definitive differences. In addition, the strategy we used in identifying cases of AKI regarding those admitted in the preceding month but remained in hospital in the index month (January or July) may have selectively involved a lower risk group regarding in-hospital mortality. However, as the majority of patients hospitalized in the index month were also admitted in the same month, the influence on the study results is supposed to be limited.

## Conclusion

We conclude that the detection rate and all-cause in-hospital mortality of AKI showed a winter predominance in patients with AKI in China. Winter appeared to be an independent risk factor for all-cause in-hospital mortality in patients with AKI. Environmental factors, including lower ambient temperature, higher humidity level, and living in temperate continental climatic regions, were independently associated with the increased risk of all-cause in-hospital mortality of AKI.

## Data availability statement

The datasets are not publicly available as they are protected by the Department of Nephrology, Peking University First Hospital. Access requests should be directed to wangjinwei@bjmu.edu.cn.

## Ethics statement

The studies involving human participants were reviewed and approved by Ethics Committee of Peking University First Hospital (2014). The Ethics Committee waived the requirement of written informed consent for participation.

## Author contributions

JL, QZ, and DZ contributed equally to the study. LY, JW, and JL proposed the concept and original design of the study. DZ and JL were responsible for the acquisition, analysis, and interpretation of the data. QZ and JL drafted the manuscript. JW, JL, and QZ provided critical revision and important intellectual content to the manuscript. LY and JW provided administrative, technical, and material support for the article. All authors contributed to the article and approved the submitted version.

## Funding

This study was supported by grants from the National Natural Science Foundation of China (Nos. 91742205, 81625004, and 82130021), the Beijing Young Scientist Program (BJJWZYJH01201910001006), the Peking University Clinical Scientist. Program by the Fundamental Research Funds for the Central Universities, and the CAMS Innovation Fund for Medical Sciences (2019-I2M5-046).

## Conflict of interest

The authors declare that the research was conducted in the absence of any commercial or financial relationships that could be construed as a potential conflict of interest.

## Publisher's note

All claims expressed in this article are solely those of the authors and do not necessarily represent those of their affiliated organizations, or those of the publisher, the editors and the reviewers. Any product that may be evaluated in this article, or claim that may be made by its manufacturer, is not guaranteed or endorsed by the publisher.

## Abbreviations

AKI: acute kidney injury
CVD: cardiac vascular disease
HT: hypertension
DM: diabetes mellitus
eGFR: estimated glomerular filtration rate
CKD: chronic kidney disease
RRT: renal replacement treatment
Scr: serum creatinine
MODS: multi-organ damage syndrome
ARDS: acute respiratory distress syndrome
DIC: disseminated intravascular coagulation.
