# Supplementary material for: Seasonal variation in the detection rate and all-cause in-hospital mortality of AKI in China: A nationwide cohort study
Source: Front Public Health. 2022 Oct 3;10:947185. doi: 10.3389/fpubh.2022.947185 (PMC9575196; doi:10.3389/fpubh.2022.947185)
Supplement: Supplementary file 1 [file Table_1.pdf]

Supplementary Table 1 Comparison between patients with or without complete data of follow-up

Number of patients	**Patients with complete data of follow-up** N=7171	**Patients missing follow-up data** N=120	***p* value**
**Demographics**[1]			
Age, years	63 (49,76)	62.5 (51,76)	0.88
Male	4652 (64.9)	90 (75)	0.02
Female	2519 (35.1)	30 (25)	
Winter	3523 (49.1)	40 (33.3)	0.001
Summer	3648 (50.9)	80 (66.7)	
Hospital-acquired AKI	3252 (45.3)	57 (47.5)	0.64
Community-acquired AKI	3919 (54.7)	63 (52.5)	
**Medical history**[2]			
Previous CVD	1973 (27.5)	33 (27.5)	0.99
Previous HT	3000 (41.8)	46 (38.3)	0.44
Previous DM	1322 (18.4)	19 (15.8)	0.47
Preexisting CKD	1747 (24.4)	21 (17.5)	0.08
Malignancy	1311 (18.3)	20 (16.7)	0.65
**Cause of AKI**			
Prerenal	5116 (71.3)	89 (74.2)	0.50
Intrinsic renal	2064 (28.8)	25 (20.8)	0.06
Post renal	631 (8.8)	16 (13.3)	0.08
**Injury factors**			
Hypovolemia	3714 (51.8)	66 (55)	0.49
Cardiac dysfunction	2018 (28.1)	36 (30)	0.65
Vascular Dilation	2346 (32.7)	34 (28.3)	0.31
Renal Vascular Constriction	870 (12.1)	18 (15)	0.34
Mechanic obstruction	117 (1.6)	1 (0.8)	0.49
Nephrotoxicity	5192 (72)	75 (62.5)	0.02
Sepsis	450 (6.2)	5 (4.2)	0.35
**AKI stage at peak**			
1	3344 (46.6)	37 (30.8)	0.001
2	1840 (25.7)	32 (26.7)	
3	1987 (27.7)	51 (42.5)	
**Comorbidities**			
MODS	866 (12.1)	22 (18.3)	0.04
ARDS	412 (5.7)	7 (5.8)	0.11
Sepsis	448 (6.2)	5 (4.2)	0.35
Shock	842 (11.7)	18 (15)	0.27
DIC	72 (1)	3 (2.5)	0.11

RRT	491 (6.8)	21 (17.5)	0.001
Length of follow-up	15 (9,24)	13 (9,22.5)	0.33

[1] The data are presented either as the mean ± SD, median (IQR) or n (%) in the following table.

[2] Abbreviations: AKI, acute kidney injury; CVD, cardiac vascular disease; HT, hypertension; DM, diabetes mellitus; CKD, chronic kidney disease; RRT, renal replacement treatment; Scr, serum creatinine. MODS, multi organ damage syndrome; ARDS, acute respiratory distress syndrome; DIC, disseminated intravascular coagulation.
